# Detection of child depression using machine learning methods

**DOI:** 10.1371/journal.pone.0261131

**Published:** 2021-12-16

**Authors:** Umme Marzia Haque, Enamul Kabir, Rasheda Khanam

**Affiliations:** 1 School of Sciences, University of Southern Queensland, Toowoomba, Australia; 2 School of Business, University of Southern Queensland, Toowoomba, Australia; Victoria University, AUSTRALIA

## Abstract

**Background:**

Mental health problems, such as depression in children have far-reaching negative effects on child, family and society as whole. It is necessary to identify the reasons that contribute to this mental illness. Detecting the appropriate signs to anticipate mental illness as depression in children and adolescents is vital in making an early and accurate diagnosis to avoid severe consequences in the future. There has been no research employing machine learning (ML) approaches for depression detection among children and adolescents aged 4–17 years in a precisely constructed high prediction dataset, such as Young Minds Matter (YMM). As a result, our objective is to 1) create a model that can predict depression in children and adolescents aged 4–17 years old, 2) evaluate the results of ML algorithms to determine which one outperforms the others and 3) associate with the related issues of family activities and socioeconomic difficulties that contribute to depression.

**Methods:**

The YMM, the second Australian Child and Adolescent Survey of Mental Health and Wellbeing 2013–14 has been used as data source in this research. The variables of yes/no value of low correlation with the target variable (depression status) have been eliminated. The Boruta algorithm has been utilized in association with a Random Forest (RF) classifier to extract the most important features for depression detection among the high correlated variables with target variable. The Tree-based Pipeline Optimization Tool (TPOTclassifier) has been used to choose suitable supervised learning models. In the depression detection step, RF, XGBoost (XGB), Decision Tree (DT), and Gaussian Naive Bayes (GaussianNB) have been used.

**Results:**

Unhappy, nothing fun, irritable mood, diminished interest, weight loss/gain, insomnia or hypersomnia, psychomotor agitation or retardation, fatigue, thinking or concentration problems or indecisiveness, suicide attempt or plan, presence of any of these five symptoms have been identified as 11 important features to detect depression among children and adolescents. Although model performance varied somewhat, RF outperformed all other algorithms in predicting depressed classes by 99% with 95% accuracy rate and 99% precision rate in 315 milliseconds (ms).

**Conclusion:**

This RF-based prediction model is more accurate and informative in predicting child and adolescent depression that outperforms in all four confusion matrix performance measures as well as execution duration.

## 1. Introduction

Depression in children is a mental health condition that affects the mind, mood and development of the children. If depression persists without treatment for a long time, children can fall behind in their usual activities such as learning [1]. Around 112,000 children and adolescents experience a severe depression in Australia, with male and female aged 4–11 years, 1.1% and 1.2%, compared to 4.3% and 5.8% between 12–17 years of age [2]. Among 10.7% of young girls and 4.5% of young boys considered suicide attempts in 2015 [2]. The actual facts for depression, which lead to issues in children’s mental health and development, must thus be discovered.

There have been several research studies on the detection of mental illness such as depression using ML algorithms in young children, university students, adults from household chores and professional duties, social network users, and so on using data of cross-sectional survey, clinical trial [3–11]. As sleep disturbances are linked to a variety of mental illnesses, sleep stages have been determined using an automatic classification system to extract complex networks by generating a weighted visibility graph to classify with kNN classifier [12]. In [9], the significance of exploring features has been emphasized in analysis that takes a long time to provide findings using RF with accuracy showed area under the curve (AUC) of 56.49%. In [3], an Random Forest (RF) classifier achieves around 92% accuracy using dataset of mental disorder patients in detection depression, bipolar disorde, obsessive-compulsive disorder and schizophrenia in the presence of a huge amount of noisy data with a significant number of missing values. The prediction accuracy of an Artificial Neural Network (ANN) model for depression detection was 97.2% in [7] with a small number of features dataset of 105 participants. Electroencephalogram (EEG) data have veen used in [8] to detect depression using K-Nearest Neighbor (KNN) classifier and the accuracy was 79.27%. DT has been applied to detect depression with a 73% accuracy using Facebook data in [11]. Relatively good results have been obtained combining textual features with the log time gap and writing hours using an RF model [5]. 95% confidence level and a 6% margin of error have been found among the data of 268 respondents [6]. Naive Bayes has provided the best accuracy of 85% in detecting depression after collecting data from 348 people [10]. According to [4], identifying stress among Bangladeshi university students has 89% accuracy rate.

Determinants of common mental health among university students have been identified by machine learning algorithms with a dataset of small number of features [13]. The missing values have been effectively added to the psychotic patient dataset for mental health detection [3, 14]. Classifying addiction patients based on substance abuse, and psychotic patients based on symptoms of various psychopathologies, such as major depression, drug-induced psychosis, schizophrenia, schizoaffective disorder, obsessive-compulsive disorder, bipolar disorder—manic episode, or bipolar disorder—mixed episode [3]. For classification, most predictive research in physical and mental health has used a variety of ML algorithms such as Random Forest (RF), Logistic Regression (LR), Support Vector Machine (SVM), Naïve Bayes (NB), k-Nearest Neighbours (kNN), Decision Tree (DT), Artificial Neural Network (ANN), Instance Based Learning (IBL), Ensemble Methods (EM) [3–6, 9–11, 13, 15–25].

Numerous studies have found that depression may be predicted with a high degree of accuracy by machine learning algorithms. However, majority of the previous work deal with small group of participants. They operate with datasets having a small number of features and, if they have large number of features, they contain a small collection of instances. Finally, they selected prediction methods at random without knowing how other algorithms performed on that particular dataset.

In this study, a large dimensional dataset of 667 features and 6310 cases has been used, with the number of classes varying widely. Furthermore, this study has investigated the performance of 32 machine learning algorithms in order to select the best approach for depression detection. There have been no research employing machine learning techniques that has used a precisely constructed dataset as Young Minds Matter (YMM) with 667 categorical features for the diagnosis of depression and 6310 number of cases [26]. The YMM is the second Australian Child and Adolescent Survey of Mental Health and Wellbeing 2013–14 collected responses from a wide, nationally representative sample of young people and their parents or care givers, including information about their mental disorders, child’s learning, social conditioning, and healthy environment [27]. The Adolescent Self-Esteem Questionnaire (ASQ) from YMM indicated that the structure had a high predictive effect on depression [26]. Using the algorithms of machine learning (ML), we present here the YMM survey results related to the sign, symptoms and cause of depression in children and adolescents, as well as its coincide.

In this research, the Boruta algorithm has been used on RF classifier for feature selection, which is one of the most influential data mining algorithms in the research community and one of the top ten data mining algorithms in terms of accuracy for removing irrelevant features as well more efficient selection of the important attributes [4, 19, 20, 28, 29]. A good analysis that demonstrates how accuracy is influenced by input data. Our aim is to use the precisely constructed dataset of YMM to discover the influencing factors and symptoms to identify depression among young children and adolescents. Because the performance of different algorithms varies depending on the scenario such as the structure of dataset, number of variable, number of input data, Tree-based Pipeline Optimization Tool (TPOTclassifier) has been applied in this research to select appropriate supervised learning models and optimize their parameters [30]. The TPOT classifier automatically detects preprocessing and modelling operators’ combinations that considerably outperformed the basic machine learning analysis by providing intelligent machine learning searches [31]. We have used the Boruta algorithm in accordance with an RF classifier to extract the most important features and apply them to appropriate supervised models to focus in particular to:

1. Present the actual signs or symptoms of depression that the children and adolescents are experiencing.
2. Evaluate the supervised models’ performances in terms of measuring Accuracy, Sensitivity, Specificity, Precision, AUC and receiver operating characteristic curve (ROC) score and execution time.
3. Show the percentage score of features of significant historical facts related to child and teen depression to learn about their family activities and socioeconomic issues that contribute to their depression.

## 2. Materials and methods

**Fig 1:**
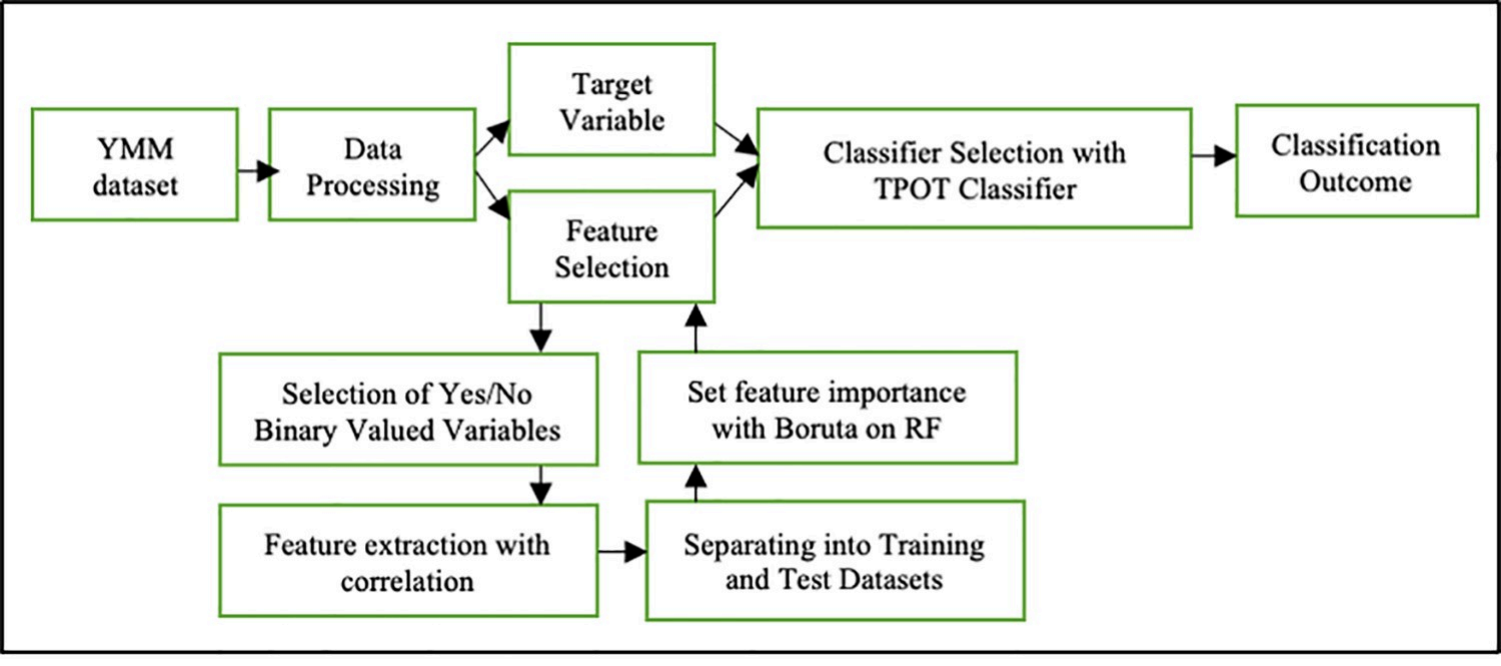
Functional pattern of the proposed method for depression detection.

Data processing has been performed to set target variable and feature selection from source dataset to evaluate the performances of ML algorithms in proposed method, shown in (Fig 1).

### 2.1 Dataset

In this study, depression has been detected using the Youth Minds Matter (YMM), a nationwide cross-sectional data by the University of Western Australia (UWA) to the Telethon Kids Institute. These data have been carried out in cooperation with Roy Morgan Research and sponsored by the Australian Department of Health between 2013 and 2014. It is the second Australian Children’s and Adolescent Survey of Mental Health and Wellbeing [27]. This dataset can be accessed by requesting to the Australian Data Archive (ADA) in the given website address (https://dataverse.ada.edu.au). YMM employed a multi-stage, area-based random sample technique. It was intended to be representative of Australian households with adolescents aged 4–17. If a family had more than one eligible child, the survey has been conducted with a single child chosen at random. A total of 6,310 parents/careers (55 percent of eligible households) of adolescents ages 4–17 were voluntarily included.

### 2.2 Data processing

#### 2.2.1 Target variable

The variables informing the status of depression confirming by doctor or mental health professional, continuing diagnosis of depression have been selected for measuring the target variable. If any of these variable’s value is true, target variable is confirmed as depressed = 1, otherwise nondepressed = 0.

#### 2.2.2 Feature selection

In our dataset, a minority class is observed to have a ‘Depressed’ class, whereas a majority class is considered to become a ‘Nondepressed’ and thus the data set is imbalanced. All variables that inform the depression confirmed by a doctor or professional mental health care professional, other than the target variable, have been removed from the training set as they were considered in the target variable.

Out of 2622 variables in the data set, 667 categorical variables of ‘Yes’ and ‘No’ values have been chosen to predict depression in this research. Using Pearson correlation of these 667 independent variables with target outcome has been measured. The variables that have low correlation with the target variable has been removed. However, correlated variables can cause misleading feature importance. For this reason, a range has been manually identified from the high correlated variables with the target variable of depression. A range of correlated values of given variables with target variable has been chosen. The best subset of input features with this range = [0.06, 0.07, 0.08, 0.09, 0.1, 0.2] has been chosen at a stage where the results of the Root Mean Square Error and coefficient of determination are the lowest and highest, respectively. The target variable of predicted depression was reported as a binary response (depressed = 1, nondepressed = 0) based on their perception of depression. The heatmap() method was used to show the correlation between the entire dataset using seaborn and matplotlib, the python data visualization library. By correlated variables, 62 variables are selected as top ranked features. These top 62 features have high correlation with target variable where Root Mean Square Error and coefficient of determination are 0.20 and 0.39 respectively.

### 2.3 Methodology

The Python 3.7.3 sci-kit-learn package has been used to produce all the machine-learning models such as RF, Boruta, TPOTclassifier, XGB, DT, GaussianNB. The RF classifier has been implemented with the Boruta algorithm in this research for unbiased and stable selection by partitioning the full dataset into training and test datasets with 62 top-ranked features. After selecting the most significant features performed by Boruta on RF, the internal_cv_score of TPOTclassifier has been considered in order to select appropriate supervised learning models and optimize their parameters [30]. The machine-learning models such as XGB, RF, DT and GaussianNB have been selected from the internal_cv_score of TPOTclassifier with class weight and threshold of 0.79, as the classification prediction is imbalanced [32]. The code has been shared publicly by the given link (dx.doi.org/10.17504/protocols.io.bzm8p49w).

#### 2.3.1 ML algorithms

*Boruta algorithm*. Boruta is a wrapper algorithm built around the RF classifier to determine the relevance and importance of features in relation to the target variable [33]. The aim of this algorithm is to remove unusable features in relation to the target variable which can create unnecessary noise [34].

*RF*. An RF algorithm has hyper-parameters indicating the number of trees and maximum tree depth considering how many interactions in the model are evaluated and the decision-making rules are the parameters [28]. It constructs numerous decision trees and combines them to get a more accurate and reliable prediction.

*TPOTclassifier*. The TPOTclassifier provides intelligent machine-learning searches which include supervised classification models, preprocessors, selection strategies and any other science-learn API-assessment estimators or transformers [31]. It can find the best parameters and model ensembles using genetic programming.

*XGB*. XGB uses gradient boosting in the random forest. The gradient boosting is achieved by giving rankings for each tree leaf and creating new trees. Depending on the performance of the previously created trees, it assigns different weights for each tree. This contrasts with traditional approaches for boosting random forests, which function by allocating the same weights to trees [35].

*DT*. Based on the divide and conquer concept, the tree is explained into class label as decision nodes or internal nodes and attributes or categories as leaf nodes. The test data or input pattern are represented through the tree’s internal nodes. The output data is presented as class label through the decision nodes of the tree [36]. Each test data through the tree’s internal nodes produces mutually exclusive and exhaustive results [37].

*GaussianNB*. Using the Naive Bayes method, GaussainNB employs the idea of maximum likelihood. The feature values are represented as vectors, and instances are assigned as class labels [38]. It calculates the probability among the values of features by a Gaussian distribution with no co variances to predict the class labels.

#### 2.3.2 Performance measure

The performance of the proposed machine learning methods has been calculated by collecting True Positive (TP), True Negative (TN), False Positive (FP), and False Negative (FN) results from the confusion matrix. The accuracy, precision, sensitivity, specificity, the score of AUC, ROC, in each ML model have been calculated using Eqs (1–4) below. XGB, DT and GaussianNB.


$$Accuracy\;Rate=\frac{TP+TN}{TP+FP+TN+FN}$$ (1)

$$Precision=\frac{TP}{TP+FP}$$ (2)

$$Sensitivity=\frac{TP}{TP+FN}$$ (3)

$$Specificity=\frac{TN}{TN+FP}$$ (4)

## 3. Experimental results

Our dataset containing 5839 nondepressed class and 471 depressed class, is completely imbalanced [39]. By separating the entire dataset into training (70% observations) and test (30% observations) datasets, the random forest classifier has been employed with the Boruta method yielding the 11 input features. In this paper, the random forest classifier has been used with Boruta algorithm for its unbiased and stable selection by splitting the entire dataset into training and test datasets accompanied by 63 top ranked features [33] shown in Table 1.

**Table 1:** Experiment findings on feature selection.

Illustration	YMM cross sectional data
Size of the training set	4417
Size of the test set	1893
Total class count	2
Total number of features in the dataset	2622
Number of subsets assessed (yes, no, unknown)	667
Number of trainings data features after correlation	62
Number of features in the optimal set	11

As the RF uses decision trees for regression and is regarded to be the best model, it is used here as the basic model. The resulted most significant 11 input features performed by Boruta around random forest classifier with class weight are shown in Table 2.

**Table 2:** Class status with most significant 11 input features.

#	Feature_ID	Depression questionnaires	Frequency Proportion	
			Yes	No
1.	Depressed	The status of depression confirming by doctor or mental health professional, continuing diagnosis of depression	7.46%	92.5%
2.	punhappy	unhappy	17.11%	
3.	PMD002	Was there a time when it seemed like nothing was fun for CHILD and [he/she] just wasn’t interested in anything?	18.78%	81.22%
4.	pmda1y	Depressed or irritable mood	28.69%	71.31%
5.	pmda2y	Diminished interest or pleasure	35.17%	64.83%
6.	pmda3y	Weight loss/gain or appetite change	43.66%	56.34%
7.	pmda4y	Insomnia or hypersomnia	37.61%	62.39%
8.	pmda5y	Psychomotor agitation or retardation	38.61%	61.39%
8.	pmda6y	Fatigue or loss of energy	40.79%	59.21%
10.	pmda8y	Thinking or concentration problems or indecisiveness	40.44%	59.56%
11.	pmda9y	Thoughts of death, suicidal ideation, suicide attempt or plan	47.10%	52.90%
12.	pmday	Presence of Any of these Five symptoms in same 2-week period	46.15%	53.85%

**Fig 2:**
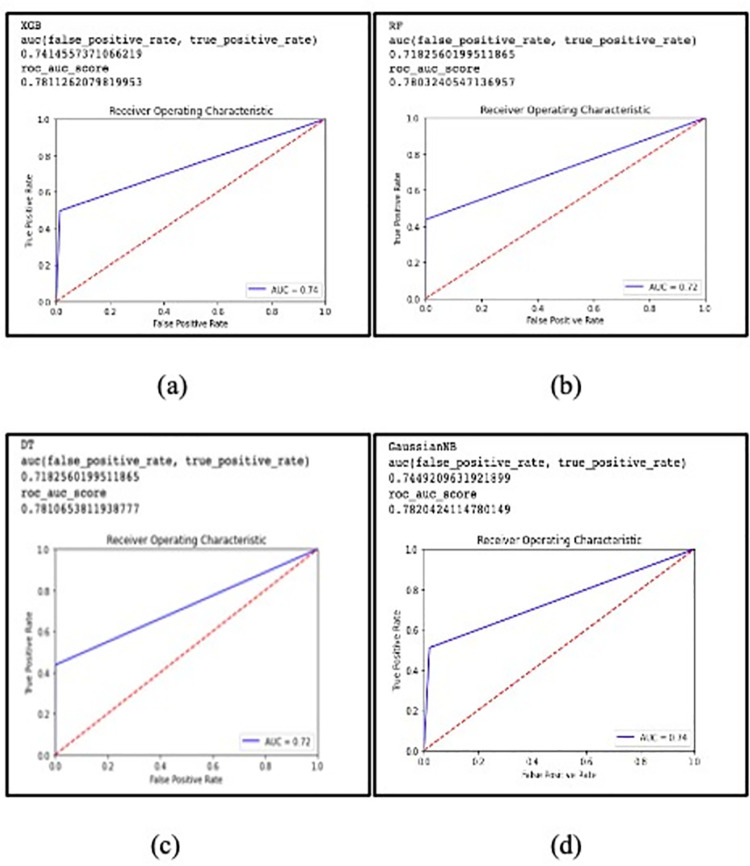
AUC and ROC scores for (a) XGB, (b) RF, (c) DT and (d) GaussianNB.

Using the sci-kit-learn module, the TPOT classifier has been applied to the training set. It obtains the scores of 32 operators. Among them, XGB, RF, DT, and GaussianNB have been selected from the internal_cv_score of TPOTclassifier to test in this research with class weight. The performance of these ML models has been reported employing four confusion matrix performance parameters such as accuracy, precision, specificity and sensitivity in Table 3, the area under curve (AUC) and receiver operating characteristic curve area (ROC) score in (Fig 2) and k-fold cross-validation techniques in Table 4.

**Table 3:** Accuracy, precision, specificity and sensitivity of XGB, RF, DT and GaussianNB.

Models	Accuracy	Precision	Specificity	Sensitivity
**XGB**	0.95	0.85	0.99	0.48
**RF**	0.95	0.99	1.00	0.44
**DT**	0.95	0.94	1.00	0.45
**GaussianNB**	0.94	0.69	0.98	0.51

**Table 4:** Result of K-Fold cross validation of depression detection.

Models	3-fold	5-fold	10-fold
**XGB**	0.9533	0.9515	0.9535
**RF**	0.9518	0.9515	0.9531
**DT**	0.9382	0.9370	0.9366
**GaussianNB**	0.9042	0.9044	0.9035

Table 3 shows the accuracy, precision, specificity and sensitivity of different ML algorithms. As we can see from the above table, the accuracies for XGB, RF, and DT are the same, but the accuracy for GaussainNB slightly less than the other ML algorithms. RF outperformed all other algorithms in predicting depressed classes with precision 99% and accuracy 95%. Since our dataset is imbalanced with 5839 nondepressed and 471 depressed class, accuracy is not a metric to indicate acceptance rates in this case. In this scenario, the ROC (AUC) score can be an optimistic measure. There is no bias in ROC analysis favouring models that perform well on the minority class at the cost of the majority class [40]. The AUC and ROC (AUC) scores of these algorithms are shown in (Fig 2). The AUC scores of XGB, RF, DT, and GaussianNB are showing the values of 0.74, 0.72, 0.72, and 0.74, respectively. These algorithms have nearly similar ROC (AUC) scores of 0.78. This ROC (AUC) value of 0.78 is within the acceptable range, and is slightly lower than the outstanding score of 0.80 [41].

**Fig 3:**
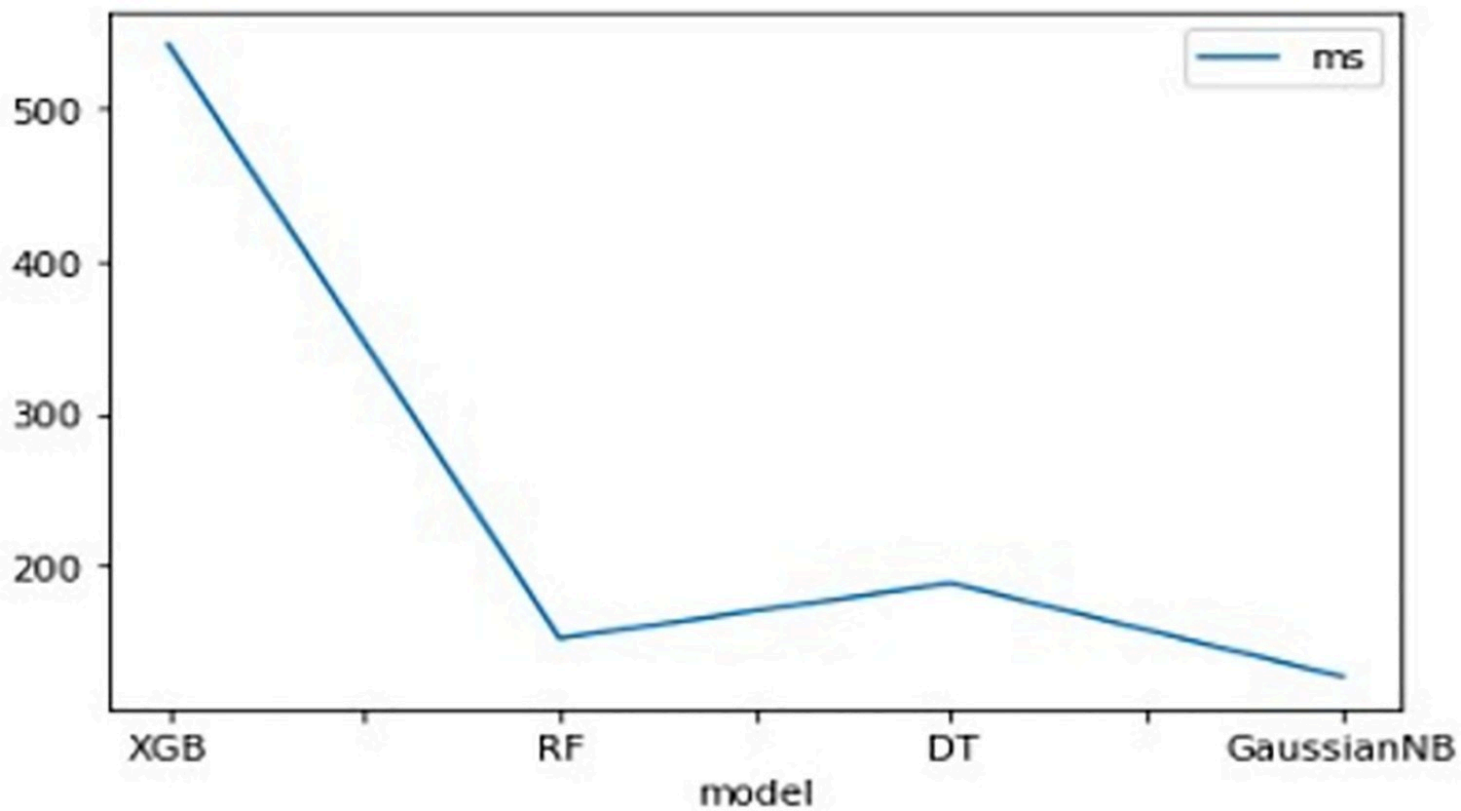
Execution times of TPOT-suggested ML models for classification.

The findings of K-fold cross-validation have been reported in Table 4 for 3-fold, 5-fold, and 10-fold repeats. Based on the accuracy scores provided in Table 3, there is very small difference in the performance of XGB and RF model in 3-fold, 5-fold, and 10-fold cross validation. Execution times for these classifications have been shown in (Fig 3) where X- axis shows the name of the models and Y- axis shows the time in milliseconds (ms).

Though the algorithms perform similarly in ROC score, and they have a very small difference in k-fold cross validation, AUC score. XGB and RF have both functioned well, with only a small variation in four confusion matrix performance parameters. RF score the best in terms of precision (99%) and specificity (100%). Moreover, the execution time for these classifiers makes a difference in selecting one of them as the best classifier. The RF exceeds in all fields in accordance with the result reported in Figs 2 and 3, Tables 3 and 4.

## 4. Discussion

It is important to understand the causative factors of depression in children and adolescents in order to detect it accurately and efficiently for their early diagnosis and development. When the parents or the involved persons in their social and academic developmental activities are unsure whether they should consult a medical expert on this issue, this research will help them make a decision to start the treatment at early stage. In this paper, we attempted to determine the specific features on the status of depression, even though the dataset was imbalanced. Class imbalance is a condition in large dataset streams that cannot be overlooked [42]. Since the number of classes fluctuate greatly, we do not want to ignore any key features that has more importance assigned to a particular class on depression analysis. Both unweighted and weighted Random Forest (RF) with Boruta has been employed in the feature selection phase, providing 8 and 11 features, respectively. These 11 features include the same 8 features that previously belonged for weighted RF. We have considered the binary answers that are the main constraints of our research, but according to the YMM questionnaire evaluation and the machine learning model prediction assessment, this feature set is clinically comparable to the major symptoms of depression [43]. As a result, using a RF model with class weight, an optimum feature set has been established for predicting depressive state. The decision path for three sample of test dataset in Table 5 shows the optimal feature set from the questionnaire using the fitted RF model to predict depression status.

**Table 5:** Prediction of depression in child and adolescence.

Unhappy	Nothing fun	Irritable mood	Diminished interest	Weight loss/gain	Insomnia or hypersomnia	Psychomotor agitation or retardation	Fatigue	Thinking or concentration problems or indecisiveness	Suicide attempt or plan	Presence of Any Five symptoms	Predicted depression
1	1	1	0	1	0	0	0	0	0	0	1
1	1	0	0	0	0	0	0	0	0	0	0
1	0	0	1	1	0	0	0	0	0	0	1
1	1	1	1	1	1	0	1	0	1	1	1
1	0	1	0	1	0	0	1	0	0	0	1
1	0	1	1	1	0	0	0	0	0	0	1

*Note: 1 = yes, 0 = no.

The sample of test dataset in Table 5 shows how RF model predicts depression in children and adolescents. In sample 1, there are yes values in unhappy, nothing fun and irritable mood and, for these values, our model is detecting depression. Again, when there are yes values in unhappy and nothing fun, it results no depression. That means, an unhappy child who has feeling nothing fun anymore cannot be addressed as depressed. In the last test sample, if unhappy children are showing no interest in anything as well losing or gaining wights, they need to be started their depression treatment as soon as possible.

It is important for machine learning model to get training data from large dimensional dataset with defined exploratory target domain to find trends, pattern and outliers to make intelligent decisions. In this research we have provided top 62 features of 667 variables of large dimensional dataset. As well, the target variable of this dataset is confirmed by doctor, mental health professional and with the status of diagnosing depression. For this reason, our model can predict depression in children and adolescents with 95% accuracy with most important 11 features in the Australian context. Moreover, the specificity of RF is 100%. Furthermore, this is an important statistic for demonstrating that RF can accurately classify the negative occurrences in the nondepressed class. Our resulted RF model provides highly accurate results for recognising both the depressed and nondepressed classes. During the analysis of these data for depression detection, certain surrounding circumstances of depressed child or adolescent become apparent in Table 6.

**Table 6:** Circumstances concerning child depression.

1.	Siblings–Adopted/foster	100%
2.	Child’s health–Poor Condition	37.14%
2.	Social Phobia	32%
3.	Caregiver: Foster Mother	27.3%
4.	Very much dislike school	25.56%
5.	House in vacant block	14.3%
6.	Taking or selling drugs	14.3%
7.	Separation Anxiety	12.1%
8.	Another parent works away	11.76%
9.	Fired from job	3.3%

Despite the fact that we have identified 11 important features for identifying child depression, the factors listed in Table 6 are noteworthy. All the children who live with their adopted or foster families are suffer from depression. One-third, 37.14% are in poor health condition. Social phobia impacted 32% of them. 27.3% of their caregivers are foster mothers. 25.56% of them do not like school. 14.3% of their residence is in a vacant block. 14.3% of them directly consume or sell drugs. Separation anxiety affects 12.1% of them. 11.76% of their another parent works in a location that is far from their home. Only 3.3% are fired from their jobs. Among all reasons of Table 6, children who have a complicated family situations as opposed to a healthy parent involvement are more likely to suffer from depression. There is evidence that children needs both parents actively involved in their life. It is extremely important in influencing a child’s learning, socialisation, and health benefits. Consequently, it can result in a more secure attachment and a sense of belonging among young children. As well, it ensures a healthy environment for a child’s upbringing.

## 5. Conclusion

Depression detection in child and adolescent is very important for the early diagnosis for their learning as well social and academic development. In this study, Random Forest (RF) has proved to be an efficient and accurate classifier to detect depression using YMM, a large dimensional dataset of mental health of children and adolescents in Australia. Moreover, the performances of all four algorithms (XGB, RF, DT, and GaussianNB) in terms of confusion matrix parameters, results of K-fold cross validation and the scores of area under curve (AUC) and receiver operating characteristic curve area (ROC), have demonstrated the TPOT classifier’s capabilities in our model. The implementation of features extraction utilizing correlation and weighted classifiers has more accurately classified the depressed individuals in the dataset. The selection of model features, high accuracy and precision rate of our RF predictive model, indicate the structure of significant predictive impact of YMM dataset on depression. As well, it contributes to the understanding of the influencing factors of depression. There are several existing predictive models for depression detection, however our model is more accurate and informative to predict child and adolescent depression in the Australian context due to its large dimensional dataset, optimal feature set and most importantly high accuracy and precision rate in prediction. It has identified the most 11 input features as questions that can be asked to children or their parents or caregivers in order to gain a preliminary understanding about whether the child is depressed or not. It can assist them in making an early diagnosis of the disease and initiating treatment at an early stage.
